# Percutaneous reduction and fixation of an intra-articular calcaneal fracture using an inflatable bone tamp: description of a novel and safe technique

**DOI:** 10.1186/1754-9493-6-6

**Published:** 2012-03-15

**Authors:** Cyril Mauffrey, James R Bailey, David J Hak, Mark E Hammerberg

**Affiliations:** 1 Department of Orthopaedics, Denver Health Medical Center, University of Colorado School of Medicine, 777 Bannock Street, Denver, CO 80204, USA; 2 Department of Orthopaedics, Naval Medical Center, San Diego, 34800 Bob Wilson Drive, San Diego, CA 92134, USA

## Abstract

Calcaneal fractures are common injuries involving the hind foot and often a source of significant long-term morbidity. Treatment options have changed throughout the ages from periods of preferred nonoperative management to closed reduction with a mallet, and more recently, open reduction and anatomic internal fixation. The current treatment of choice; however, is often debated, as open management of these fractures carries many risks to include wound breakdown and infection. A less invasive form of surgical management through small incisions, while maintaining the ability to obtain joint congruency, anatomic alignment, and restore calcaneal height and width would be ideal. We propose a novel form of fracture reduction using an inflatable bone tamp and percutaneous fracture fixation. Preoperative planning and experienced fluoroscopy is crucial to successful management using this method. Although we achieved successful radiographic outcome in this case, long-term functional outcome of this technique are yet to be published.

## Background

Two thirds of hind foot fractures involve the calcaneus [1]. The treatment of choice for intra-articular fractures is still debated and a number of trials have flourished in recent years to address this particular issue [2,3]. Possible treatments options for a depressed intra articular fracture include limb elevation with application of a bulky jones dressing, open reduction internal fixation with plate and screws or percutaneous reduction and fixation using screws. Other techniques using fine wire external fixation have also been described [4]. The goal of the treatment of intra-articular displaced fractures is to focus on the anatomical reduction of the articular surface, avoid complications, and correct the length, width and angulation of the tuberosity. The open reduction and internal fixation technique allows the operator to view the articular surface directly during the reduction and fixation process but the high rate of wound breakdown and infection (15-40%) is a concern [5,6]. Percutaneous techniques use, by definition, a smaller incision but the reduction is often challenging and inadequate. We propose a novel form of reduction using an inflatable bone tamp. This technique has been described in the tibial plateau [7,8] but very few papers are available for its application in the calcaneus [9].

## Case report

### Pre-operative planning

**Figure 1:**
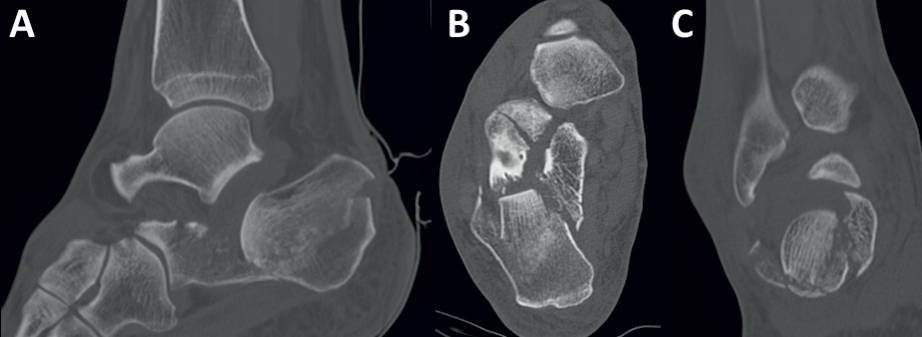
**CT scan images of the sagittal (A), axial (B), and coronal view (C) of the fracture at the point of depression of the posterior facet**.

**Figure 2:**
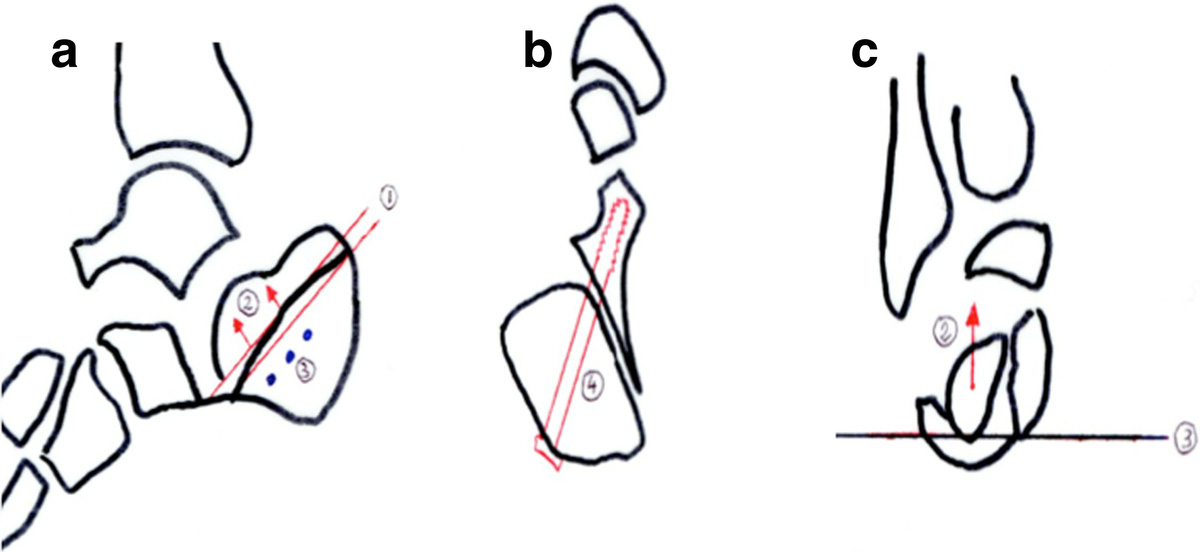
**Sagittal cut (A) of the pre operative planning with the cannula insertion (1), the direction of vector for the balloon inflation (2) and the K-wires for balloon support (3); axial cut (B) of the preoperative planning through the sustentaculum fragment**. The number 4 shows the preoperative planning for the screw direction. Coronal cut (**C**) of the preoperative planning putting in evidence the fragment to elevate and the direction for the balloon inflation (2) as well as the K-wires (3).

The preoperative planning is critical. A computerized tomography is essential to visualize the fragments involved (Figure 1A, B, C). A number of issues need to be assessed during the planning phase. The first aspect is the identification of the fracture lines and the position of the depressed articular fragment. The former will determine screw placement following reduction maneuver, while the latter will determine the path of the cannula as well as the inflatable bone tamp position in order to obtain the optimal force direction for anatomical reduction. We use templating paper to trace the sagittal, axial and coronal images from the CT scan using the cuts where the articular fragment is most depressed (Figure 2). We use the sagittal view to determine the ideal path for the cannula and drill bit through which the balloon will be inserted (Figure 2a). We also use this view to plan for Kirschner wire insertion from lateral to medial beneath the cannula to create a support for the balloon to inflate proximally and elevate the fragment (Figure 2a). The combination of the angle of cannula insertion and the angle at which the 3 wires are inserted will determine the vector of reduction of the articular fragment (Figure 2a). We use the axial view to assess the direction of the screw fixing the sustantaculum tali fragment. This is a screw from posterolateral to anteromedial (Figure 2b). The coronal view helps us determine how distal must the Kirschner wires be inserted and their angulation in relation to the articular fragment above them to create the appropriate vector of reduction when the balloon is inflated over these wires (Figure 2c).

### Patient positioning

We use a radiolucent table. The optimal positioning of the patient is prone with foam padding placed under both knees and a bump under the affected leg to achieve lateral fluoroscopy images without having to move the unaffected leg. Intravenous antibiotics are given at induction before the tourniquet is inflated. The leg is prepared up to the mid-thigh with Chlorhexidine solution and draped in sterile manner. With a sterile marker pen we highlight the Achilles tendon insertion on the calcaneus.

A clear adhesive dressing is applied around the foot and ankle and infiltration of 2 ml of 0.25% Marcaine with 1/200,000 of adrenaline is infiltrated into the area of incision down to the periosteum to reduce postoperative pain and intraoperative bleeding.

### Procedure

**Figure 3:**
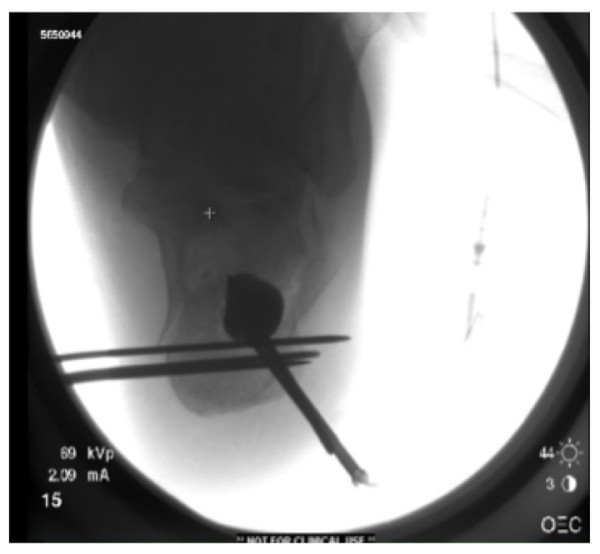
**Harris view with the inflated balloon and the three K wires below the inflatable tamp**.

**Figure 4:**
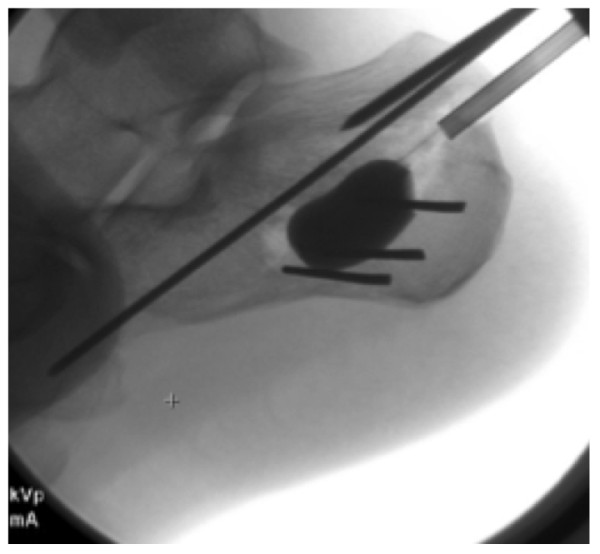
**Sagittal view of the inflated balloon with the three K wires providing support from beneath**.

**Figure 5:**
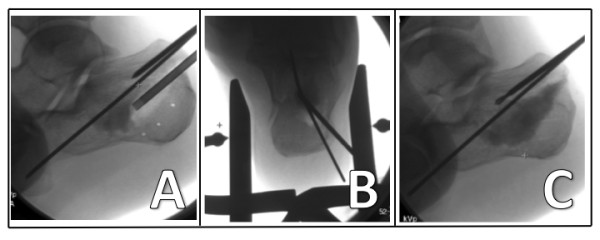
**Sagittal view (A) showing a cannula in situ with calcium phosphate insertion and a K-wire temporarily stabilizing the reduced articular fragment**. Two large osteotomes wrapped in towels are used with peri-articular clamps to correct the tuberosity varus angulation and width (**B**). Sagittal view (**C**) with K wire temporary fixing the reduced articular fragment.

**Figure 6:**
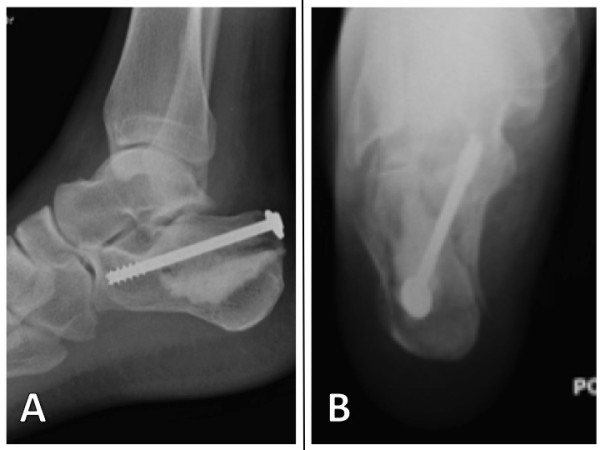
**Post-operative sagittal view (A) and Harris view (B) showing the calcium phosphate and a 7.5 mm cannulated screw fixing the tuberosity to the sustentaculum tali via the articular fragment**.

The skin is marked with a 3 cm curved incision on the posterolateral aspect of the proximal third of the calcaneal tuberosity. The skin incision is made and subcutaneous fat dissected down to the lateral border of the Achilles tendon insertion. A guide wire is used to determine the entry point for the cannula as determined by the preoperative templating. The cannula is inserted through the proximal cortex and a drill bit used through the cannula to create a path for the cannula to lie beneath the articular fragment ensuring to leave a thick portion of subchondral cancellous bone to elevate together with the articular surface. Both a lateral and Harris view are checked to confirm the adequate position of the tip of the cannula in relation to the depressed articular fragment. At this stage three, 2-mm Kirshner wires are inserted from lateral to medial just beneath the cannula to create a rafting support for the balloon inflation. The drill bit is removed and the balloon inserted through the cannula. The device we use is known as the Inflatable Bone Tamp (IBT, Kyphon/Medtronic, Inc, Sunnyvale, CA). It is comprised of three biocompatible parts: a proximal luer fitting, a central catheter, and a distal inflatable tip with radiopaque markers. Inflation of the balloon is achieved by using an external inflation syringe filled with radiopaque dye (i.e. Omnipaque™) connected to the proximal luer-lock connection. This inflation syringe measures the volume (cc) and the pressure (psi) of the inflation. These characteristics allow the device to be used in any bone simply as a conventional bone tamp or as a percutaneous bone tamp with fluoroscopic guidance. The inflatable bone tamp is FDA-approved for use as conventional bone tamps for the reduction of fractures and/or creation of a void in cancellous bone in the spine, hand, tibia, radius and calcaneus. Two radiolucent markers on the balloon allow the operator to determine its ideal position in the sagittal plane. The balloon is inflated while the operator keeps a close eye on the pressure monitor (in ppi) and the volume. An inflation pressure of around 250 ppi should suffice to elevate the articular fragment. The radiopaque dye contained in the balloon allows the surgeon direct visualization of its position in space. The volume is noted, as this will need to be replaced by an equivalent volume of bone substitute. The operator then confirms the adequacy of reduction of the articular surface on a lateral and a Harris view (Figure 3). One could potentially also make a small lateral incision and access the subtalar joint reducing the calcaneal posterior facet under direct vision. We prefer to keep the procedure as minimally invasive as possible and find that good lateral and Harris views are sufficient to confirm reduction. The next step is to temporary stabilize the articular fragment of the posterior facet and we favor insertion of a 2-mm K-wire from posteromedial into the calcaneo-cuboid joint going through the newly reduced fragment (Figure 4). Following this maneuver, the balloon is deflated and the cannulas removed. The bone void left by the balloon expansion and the cannula track is quantified by the volume of the balloon at its maximal distension. This void can be filled in with autologous bone graft, allograft, bone substitute or alternatively cement. We prefer to use calcium phosphate in an injectable form (Hydraset, Stryker) (Figure 5). We place a cannula under fluoroscopy control so that the tip is placed at the base of the void for retrograde filling. The bone substitute is left to harden to a state that it can be drilled after approximately 12 minutes. We then place one or two 7.3 mm partially threaded cannulated screws following the track identified in the preoperative templating. In the case highlighted here, we had a depressed posterior facet and a separate sustentaculum tali fragment. We opted to fix the posterior facet to the tuberosity with one 7.3 mm screw ending in the sustentaculum fragment. We used a periarticular clamp placed from medial to lateral to correct the varus deformity, reduce the lateral wall comminution and reduce the sustentacular fragment during screw insertion. Our technique is to wrap two broad osteotomes in a towel and place them on the medial and lateral sides of the hind foot. A periarticular clamp is placed over these two osteotomes and the deformities identified on the Harris view (coronal plane) can be corrected (Figure 5b). These Harris views are obtained to ensure fracture reduction, articular congruence, and to ensure the calcium phosphate has not extravasated. Flat plates are checked prior to the patient waking up to make sure that the screw position is adequate, the fracture reduced and the calcium phosphate in the void (Figure 6).

### Closure

**Figure 7:**
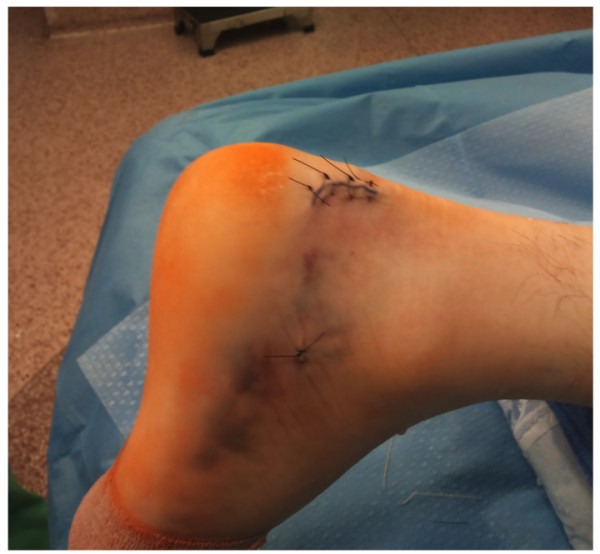
**Clinical postoperative photograph of the right foot with a closed posterolateral incision**.

The wound is thoroughly irrigated and closed with vicryl 2.0 and Nylon 3.0 to skin (Figure 7). The patient is placed in a confortable splint with the foot in neutral position.

### Postoperative instructions

We do not check our wounds until removal of sutures on the 14^th ^day post op. We leave the wounds dry and covered. When the sutures are removed, the leg is placed in a protective postoperative boot and the patient remains non-weight bearing for a total of 10 weeks, or until the fracture is radiologically and clinically healed. Physical therapy is important in the early phases to allow active and passive knee, ankle and forefoot range of motion and prevent stiffness.

### Pitfalls and difficulties

The operator should be aware of pitfalls and difficulties when performing this technique. During the reduction maneuver, the balloon can burst, leaving the radio-opaque dye in the sub articular region. This can occur if the balloon encounters a very sharp bone fragment. Should this occur, the surgeon should irrigate the bone void via the cannula using normal saline and reinsert a balloon. Another possible difficulty is that the balloon does not inflate evenly and the articular fragment does not elevate. This has been described as the trapdoor phenomenon in tibial plateau fractures [10]. This phenomenon occurs when the compression in the medial to lateral plane prevents the reduction of the articular fragment. It is therefore important not to apply a clamp to reduce the lateral wall or the sustentaculum tali fragments until the articular fragment has been elevated to its anatomical position. Finally, the operator should understand the risk of having a liquid form of calcium phosphate injected into a bone void with potential extravasation into the subtalar joint or through the lateral wall. This risk can be limited by the correct timing of injection of the calcium phosphate so that it is not too runny but also by regular fluoroscopy so as to stop the injection if there is extravasation.

## Conclusions

We present one of the first in vivo descriptions of the use of an inflatable bone tamp for intra-articular calcaneal fractures. It is crucial to select the right patients with a fracture that is both amenable to reduction by inflation plasty and with comminution not to severe as to have extravasation of a liquid bone substitute in the joint. The ideal fracture is one with a large depressed articular fragment. The preoperative planning is also critical to determine the ideal angle of the cannula insertion and plan for the vector of push during the balloon inflation phase. This will define the location of insertion of the wires to provide support during the inflation phase. Preoperative planning also serves to identify the angle and direction of the screw that will support the articular fragment and fix the sustentaculum tali fragment. The long-term advantages of this technique are yet to be published and we have no data on long-term functional outcome.

## Consent

The patient fully agreed with publication of this case report, including the publication of medical data, radiological imaging, and intraoperative pictures. Written informed consent is available to the Editor-in-Chief upon request.

## Competing interests

The authors declare that they have no competing interests.

The views expressed in this article are those of the authors and do not reflect the official policy or position of the Department of the Navy, Department of Defense, or the United States Government.

## Authors' contributions

CM, JRB, DJH designed the case report. CM and JRB performed the surgical procedures in this patient. CM drafted the first version of the manuscript. All authors contributed and approved the final version of the manuscript.
